# The Association Between Geriatric Nutritional Risk Index and Readmission Within Six Months in Elderly Heart Failure Patients: A Retrospective Cohort Study: Geriatric Nutritional Risk Index for Heart Failure Readmission Within 6 Months

**DOI:** 10.1155/2024/5692215

**Published:** 2024-10-24

**Authors:** Guoxia Dong, Zhihua Li

**Affiliations:** ^1^Department of General Practice, Affiliated Hospital of Jining Medical University, Jining 272029, China; ^2^Department of Cardiology, Affiliated Hospital of Jining Medical University, Jining 272029, China

**Keywords:** elderly, geriatric nutritional risk index (GNRI), heart failure (HF), malnutrition, readmission

## Abstract

**Background:** The geriatric nutritional risk index (GNRI) is a valuable tool that may predict the prognosis of elderly patients with heart failure (HF). Malnutrition and low GNRI scores have been associated with a higher risk of hospitalization and mortality. This study aimed to investigate the association between GNRI and 6-month readmission for HF in elderly Chinese patients.

**Materials and Methods:** The study utilized data from hospitalized HF patients by combining electronic medical records from the PhysioNet restricted health data database with external outcome data. In our study, we used the GNRI as the independent variable and assessed its association with the risk of readmission within 6 months. The main analytical methods were multivariable Cox regression, stratified analysis with interaction, threshold effect analysis, and Kaplan–Meier survival curves.

**Results:** This study involved 767 elderly HF patients, 61.3% of whom had malnutrition. In the threshold analysis, HF patients' 6-month readmission risk was significantly reduced with increasing GNRI, with a hazard ratio (HR) and 95% confidence interval (CI) of 0.99 (0.97.1). Malnutrition group was associated with a higher risk of readmission within 6 months for HF patients in analyses that were controlled for confounding factors, with HRs and their 95% CI of 1.17 (0.99, 1.38), 1.18 (1, 1.4), and 1.44 (1.08,1.93), respectively. Subgroup analysis showed that GNRI levels had a consistent impact on outcome events, unaffected by covariates.

**Conclusions:** GNRI was negatively correlated with the outcome event of readmission within 6 months in elderly HF patients. Malnutrition group showed a higher risk of readmission within 6 months.

## 1. Introduction

Heart failure (HF) remains a global public health issue and is one of the leading causes of hospitalization in elderly people [1–3]. There is an obvious surge in the prevalence rate of HF from 0.9% in individuals aged 55–64 years to 17.4% in those over the age of 85 years [4]. Aging population in China is increasing continuously and has entered a phase of demographic aging. Readmission rates of HF within 6 months were reported to reach as many as 45% [5]. The economic strain of HF is anticipated to rise unless there is a reduction in hospitalization rates, due to demographic changes leading to a higher total number of individuals living with HF [6]. In consideration of the features and poor prognosis of older HF patients, early recognition of elderly HF patients at high risk of readmission is of paramount importance.

Malnutrition is a common complication in elderly population with HF [7]. Malnutrition in HF is associated with unfavorable outcomes [8]. Several clinical research studies have reported that malnutrition is a powerful independent prognostic factor of mortality in individuals with HF. Geriatric nutritional risk index (GNRI) is an uncomplicated and well-established screening tool for nutrition-related risk for elderly populations and chronically ill patients [9]. It can predict the prognosis in patients with HF [10]. Lower GNRI scores are associated with an increased risk of stroke in elderly patients with hypertension [11]. In the oldest-old patients with acute coronary syndrome, a low GNRI independently predicted the risk of all-cause mortality [12]. Within the population of patients in the cardiac intensive care unit, an elevated risk of in-hospital mortality was significantly associated with a diminished GNRI [13]. The calculation of GNRI is based on both body mass index (BMI) and serum albumin level. It includes two parameters, body weight and serum albumin, which are easy to measure and obtain during regular check-ups [14]. GNRI, the nutritional status management score, the prognostic nutritional index (PNI), and controlling nutritional status (CONUT) are commonly utilized objective criteria for evaluating nutritional status, in elderly individuals with acute decompensated HF. Compared to the other two objective nutritional indexes, GNRI is the most effective predictor of readmission for worsening HF within 1 year after discharge [15]. The purpose of this research was to investigate the association between GNRI and readmission for HF within 6 months in older Chinese adults.

## 2. Materials and Methods

### 2.1. Data Source

This retrospective cohort study included patients who were chosen from a publicly accessible Chinese population database available on PhysioNet (https://physionet.org/content/heart-failure-zigong/1.3/) [16, 17]. Author Dong has successfully completed the educational course provided by the National Institutes of Health and was granted access to the database (certification number: 58380637). Institutional Review Board approval was obtained (Approval Number: 2020-010) prior to the start of the study. Zigong database contains data regarding demographic characteristics, comorbidities, laboratory reports, results of imaging, final outcomes, records of care, and therapeutic drugs for patients suffering from HF. As the data were sourced from the international public database PhysioNet, the ethics committee of our institution did not need to grant approval for this study.

### 2.2. Study Population

We retrospectively analyzed records of 2008 individuals with HF, who were hospitalized at the Fourth People's Hospital in Zigong, Sichuan, China, from December 2016 to June 2019.

### 2.3. Subject Selection

**Figure 1:**
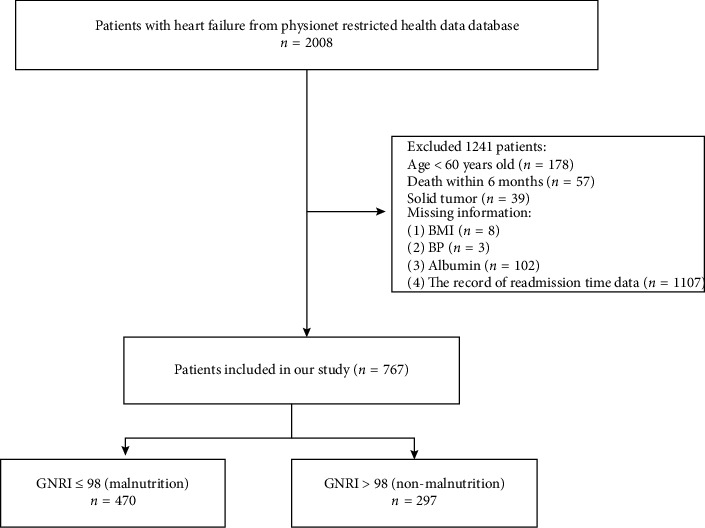
Flowchart of patient selection. BMI, body mass index, BP, blood pressure, GNRI, geriatric nutrition risk index.

Elderly patients with HF who had available records of readmission within 6 months and the corresponding readmission time were selected. HF was defined following the guidelines of the European Society of Cardiology (ESC) [18]. Nurse manually entered their demographic data into a digital patient record system that integrates the Ninth Revision of the International Classification of Diseases after admission to hospital. The exclusion criteria were as follows: (1) age < 60 years (*n* = 178), (2) death within 6 months (*n* = 57), (3) solid tumor (*n* = 39), and (4) missing information: BMI (*n* = 8), BP (*n* = 3), albumin (*n* = 102), and the record of readmission time data (*n* = 1107). Finally, a total of 767 patients who completed a 6-month follow-up were selected (Figure 1). Malnutrition was defined as a GNRI of 98 or less, and patients were categorized into two groups based on their GNRI: the malnutrition group (GNRI ≤ 98) and the nonmalnutrition group (GNRI > 98).

### 2.4. GNRI

The GNRI was calculated by applying the following formula: (1.489 × serum albumin [g/L]) + (41.7 × weight [kg]/ideal body weight [kg]) [19]. Ideal body weight = 22 × the square of the height in meters.

### 2.5. Study Variables

In our study, GNRI was the exposure variable, and HF readmission within 6 months was the main endpoint variable. Furthermore, we obtained extra information, including gender, age, systolic blood pressure, weight, height, BMI, NYHA classification, COPD, chronic kidney disease, diabetes, urea, uric acid, potassium, sodium, albumin, lactate, hemoglobin, brain natriuretic peptide, Charlson Comorbidity Index (CCI) score, and drugs.

### 2.6. Endpoints and Follow-Up

The main outcome was readmission within 6 months postdischarge. All participants were observed for 6 months to obtain the admission. When required, medical records were checked to confirm the results.

### 2.7. Statistical Analysis

Data description was performed for every participant. Continuous variables are presented in the form of mean ± SD for normal data distribution and median (minimum, maximum) and interquartile range (IQR) for skewed data distributions. Categorical variables were reported as percentages or frequencies. We applied the *T*-test, Kruskal–Wallis test, and chi-square test for the comparison of normally distributed, nonnormally distributed, and categorical continuous variables, respectively. We used the Kaplan–Meier analyses to determine survival curves. The research participants were categorized into the following two groups according to GNRI levels: the malnutrition group (GNRI ≤ 98) and the nonmalnutrition group (GNRI > 98).

Multivariable Cox regression analyses were employed to determine the independent association between GNRI and HF 6-month readmission. The covariates were adjusted based on three criteria: (1) in univariate analyses, *p* values lower than 0.1; (2) variables whose odds ratios show a change of at least 10% after being incorporated into the model; and (3) in consideration of clinical knowledge. Cox regression models were utilized to calculate hazard ratios (HRs) and 95% confidence intervals (95% CIs) in order to analyze the association between GNRI and HF 6-month readmission. Multivariable models were adjusted as follows: (1) Model 1: adjusted for gender and age; (2) Model 2: Model 1 with further adjustment for diabetes, CKD, MI, COPD, NYHA classification, and CCI score; and (3) Model 3: Model 2 with an adjustment for creatinine, uric acid, potassium, sodium, hemoglobin, lactate, brain natriuretic peptide, and diuretics. The examination of interactions among independent variables involved multiple covariance tests with variance inflation factor (VIF).

Subgroup analyses were carried out using stratified Cox regression models. The likelihood-ratio tests were used to assess modifications and interactions of subgroups. We performed a two-tailed test. When *p* < 0.05, the statistical differences were identified as statistically significant. To address missing data, we employed the R mice procedure, which utilizes multiple imputation with 5 replications and a chained equation approach method. Each of the analyses was carried out using statistical software (https://www.R-project.org, The R Foundation) and Free Statistics software Version 1.9.

## 3. Results

### 3.1. Baseline Characteristics

This retrospective cohort study ultimately consisted of 767 elderly patients with HF, of which 39.5% were over 80 years of age and 41.3% were male, and the proportion of NYHA classification was 12.8% (Class II), 51% (Class III), and 36.2% (Class IV), respectively. Figure 1 displays the flowchart detailing the patient selection process.  Table 1 presents the fundamental characteristics of all individuals in the malnutrition group and the nonmalnutrition group. The malnutrition group included 470 (61.3%) patients, compared to 297 (38.7%) patients in the nonmalnutrition group (Table 1). Baseline data indicated that there were no significant differences in variables (gender, NYHA classification, and CCI score) between the two groups (Table 1). Patients were more likely to have higher malnutrition in age greater than 80 years. Patients in the malnutrition group had lower SBP, hemoglobin, and GNRI and higher NT-proBNP and furosemide injection application (Table 1).

### 3.2. GNRI Levels and Outcome Events

After 6 months, 598 (78%) participants had an endpoint outcome, with 376 and 222 endpoint events occurring for the two groups, respectively. The malnutrition group had a higher incidence proportion of 80% compared to the nonmalnutrition group's rate of 74.7% (Table 1). GNRI and HF 6-month readmission rate showed a linear association (*p*=0.262 for nonlinearity, Supporting Figure S1). The malnutrition group was linked to a higher readmission risk within 6 months.

### 3.3. Sensitivity and Subgroup Analysis

**Figure 2:**
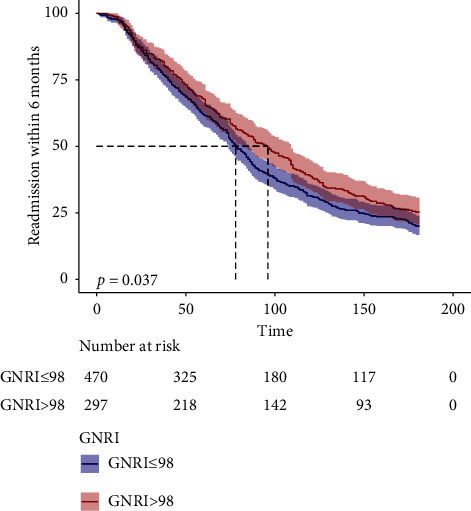
Readmission of HF within 6 months among groups was plotted over time on a Kaplan–Meier survival curve.

**Figure 3:**
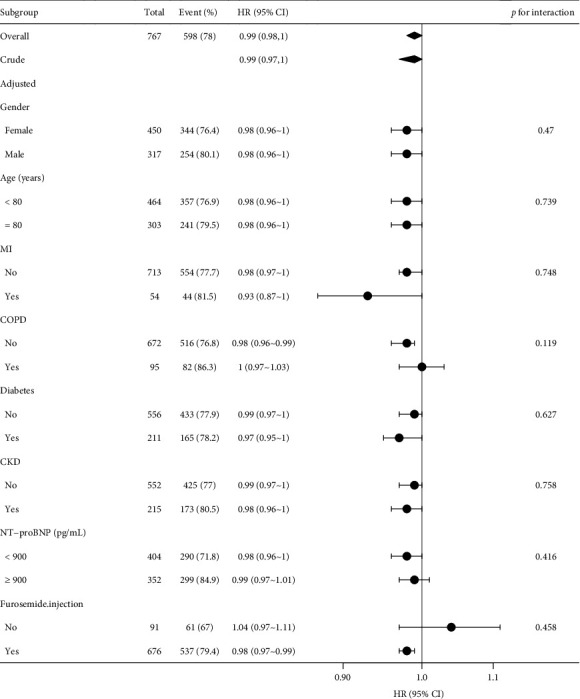
Subgroup analysis of readmission outcome events.

The research further investigated the HRs and their corresponding 95% CIs linked to different GNRI levels and their impact on the likelihood of readmission within 6 months for elderly HF patients. The association between GNRI levels and the risk of HF readmission within 6 months persisted adjusted by the three models (Model 1: HR = 0.99, 95% CI 0.98–1, *p*=0.016; Model 2: HR = 0.99, 95% CI 0.98–1, *p*=0.022; Model 3: HR = 0.99, 95% CI 0.97–1, *p*=0.038). The statistical findings showed that following the categorization of patients based on GNRI levels and subsequent multifactorial regression analysis employing the three models, those in the malnutrition group were identified as having a heightened risk of adverse outcomes (Model 1: HR = 1.17, 95% CI 0.99–1.38, *p*=0.072; Model 2: HR = 1.18, 95% CI 1–1.4, *p*=0.056; Model 3: HR = 1.44, 95% CI 1.08–1.93, *p*=0.013) (Table 2). Kaplan–Meier estimates of rehospitalization showed that the malnutrition group had a substantially higher risk of 6-month readmission for HF (Figure 2). GNRI levels were found to have a stable effect on outcome events in the subgroup analysis, and this effect was not influenced by covariates (gender, age, diabetes, CKD, MI, COPD, NYHA classification, CCI score, diuretics, creatinine, uric acid, potassium, sodium, hemoglobin, lactate, NT-proBNP, and diuretics) (Figure 3).

## 4. Discussion

The evaluation of the nutritional status in our study was conducted using the GNRI. The results suggested that GNRI was negatively correlated with HF readmission within 6 months. To control for potential confounders, the correlation between GNRI and HF readmission within 6 months was analyzed by three Cox regression models. We established three Cox regression models to analyze the association between GNRI and HF readmission within 6 months. In fully adjusted Model 3, the effect value is 0.99 (0.97–1). This means that for each additional unit of GNRI, the risk of HF readmission within 6 months decreased by 1%. The results on 767 patients with HF showed that malnutrition in elderly individuals with HF played a significant role in influencing readmission within 6 months and might be employed as an indicator for determining the prognosis of patients. The robustness of the relationship between GNRI and HF readmission within 6 months was evident in both the stratified and sensitivity analyses. In the management of HF patients, even a small reduction in risk can have a significant impact on the long-term health of patients and the burden on the healthcare system, which could mean significant healthcare resource savings and improved quality of life for patients on a large scale. Malnutrition is a potentially modifiable risk factor for reducing the risk of death in elderly patients with diabetes mellitus [20].

Clinical indicators of nutritional status, such as the GNRI, CONUT score, and PNI, are extensively used. All three indexes have proven useful to predict the prognosis of patients with HF [21–23]. Yoshihiro Sato et al. found that GNRI is the most predictive of rehospitalization for elderly HF patients [24]. Our study focuses on a specific demographic: elderly HF patients in China, a group that has been relatively understudied globally, with the 6-month readmission as the primary endpoint event. The average period to readmission was 91.9 ± 59.1 days for HF patients in the malnutrition group and 101.3 ± 60.4 days for those in the nonmalnutrition group, indicating that low GNRI levels were associated with long-term outcomes (Table 1).

Several meta-analyses of prospective studies and retrospective designs have shown that older HF patients who have lower GNRI levels are independently associated with increased all-cause mortality and major cardiovascular events. Using GNRI to evaluate nutritional status could enhance risk stratification for elderly HF patients [25, 26]. Nutritional risk serves as a crucial predictor of adverse hospital outcomes postsurgery [27]. Combining GNRI with simplified nutritional index can predict the risk of major adverse cardiovascular events in older adults with acute coronary syndromes within 1 year [28]. Consistent with our results, in comparison with activities of daily living (ADL) ability or lower limb muscle strength, a nutrition status assessment using GNRI showed a stronger predictive value. HF patients who exhibited a low GNRI discharged from the hospital may face an unfavorable prognosis after 1 year [29]. Sato et al. found that the GNRI provided the highest accuracy in predicting readmission for worsening HF 1 year after discharge from the hospital among the elderly individuals with acute decompensated HF [30]. Nonetheless, the findings of the study were based on small sample sizes and the generalizability of the findings to China is uncertain.

The generalizability of the research is important, the same as other clinical investigations, and the researchers established the scope of this research in advance. As the patients are from southwest China and due to the small sample size, our results may not be entirely representative of the overall older HF individuals. Additionally, some patients were excluded because of incomplete data, and for this reason, the internal validity of our results may be compromised in terms of generalizability. In designing future trials, a solution that could be considered is to enroll a larger and more patient population, gather the most extensive medical information, and stratify these data to enhance generalizations of results.

It is important to note that our study has certain limitations. The investigation was, first, a retrospective, single-center study. Consequently, it is not feasible to completely rule out the possibility of unintentional selection bias among the patients. Second, because patient's chronological age was described in range of ages, we have no information on specific age data, which hindered a precise analysis of the age effect on patient readmission. Third, although possible covariates are adjusted in the Cox regression model, the influence of other unmeasured confounding factors could not be ruled out. Fourth, in our subsequent research, we will consider incorporating other nutritional assessment tools such as the Subjective Global Assessment (SGA), Mini-Nutritional Assessment (MNA), or serum nutritional biomarkers to provide a more comprehensive evaluation of patients' nutritional status. Last, our study lacked background information on exercise habits and dietary patterns.

## 5. Conclusions

We discovered that GNRI was negatively correlated with the occurrence of readmission within 6 months in individuals suffering from HF in a hospitalized HF cohort in southern China. Malnutrition group showed a higher risk of readmission within 6 months. Nevertheless, subsequent prospective investigations will be necessary to verify the current findings.

## Figures and Tables

**Table 1:** Baseline characteristics of selected participants.

Variables	Total	GNRI ≤ 98 (malnutrition)	GNRI > 98 (nonmalnutrition)	*p* value
*N*	767	470	297	
Gender, *n* (%)				0.188
Female	450 (58.7)	267 (56.8)	183 (61.6)	
Male	317 (41.3)	203 (43.2)	114 (38.4)	
Age, *n* (%)				< 0.001
< 80 years	464 (60.5)	249 (53)	215 (72.4)	
≥ 80 years	303 (39.5)	221 (47)	82 (27.6)	
SBP (mmHg)	129.7 ± 23.6	127.7 ± 23.4	132.9 ± 23.6	0.003
Weight (kg)	52.1 ± 10.7	47.7 ± 7.7	59.2 ± 11.0	< 0.001
Height (m)	1.6 ± 0.1	1.6 ± 0.1	1.6 ± 0.1	0.559
BMI (kg/m^2^)	21.2 ± 3.8	19.3 ± 2.4	24.2 ± 3.8	< 0.001
NYHA classification, *n* (%)				0.184
II	98 (12.8)	56 (11.9)	42 (14.1)	
III	391 (51.0)	232 (49.4)	159 (53.5)	
IV	278 (36.2)	182 (38.7)	96 (32.3)	
CCI score, *n* (%)				0.673
≥ 3	215 (28.1)	138(29.3)	77 (26)	
Diabetes, *n* (%)				0.379
Yes	211 (27.5)	124 (26.4)	87 (29.3)	
COPD, *n* (%)				0.281
Yes	95 (12.4)	63 (13.4)	32 (10.8)	
CKD, *n* (%)				0.386
Yes	215 (28.0)	137 (29.1)	78 (26.3)	
MI, *n* (%)				0.792
Yes	54 (7.0)	34 (7.2)	20 (6.7)	
Readmission, *n* (%)	598 (78.0)	376 (80)	222 (74.7)	0.087
Hemoglobin (g/L)	111.8 ± 23.6	107.0 ± 23.7	119.3 ± 21.3	< 0.001
Uric acid (mg/dL)	489.8 ± 173.2	488.0 ± 173.5	492.7 ± 172.8	0.715
Potassium (mmol/L)	4.0 ± 0.7	4.1 ± 0.7	4.0 ± 0.7	0.073
Sodium (mmol/L)	138.1 ± 4.9	137.8 ± 5.0	138.5 ± 4.8	0.044
Lactate (mmol/L)	1.8 (1.5, 2.6)	1.8 (1.5, 2.6)	1.9 (1.4, 2.4)	0.469
Albumin (g/L)	36.9 ± 4.6	35.0 ± 3.9	39.9 ± 4.1	< 0.001
Furosemide injection, *n* (%)				0.025
Yes	676 (88.1)	424 (90.2)	252 (84.8)	
Furosemide tablet, *n* (%)				0.83
Yes	656 (85.5)	403 (85.7)	253 (85.2)	
Spironolactone tablet, *n* (%)				0.603
Yes	716 (93.4)	437 (93)	279 (93.9)	
NT-proBNP (pg/mL), median (IQR)	815.9 (325.4, 1765.1)	951.1 (360.7, 1970.1)	619.7 (282.6, 1387.2)	< 0.001
Urea (mmol/L), median (IQR)	8.6 (6.2, 12.2)	8.9 (6.2, 12.9)	8.2 (6.0, 11.5)	0.068
GNRI	95.2 ± 10.1	88.8 ± 6.3	105.2 ± 6.2	< 0.001
Time interval to readmission	95.5 ± 59.8	91.9 ± 59.1	101.3 ± 60.4	0.033

*Note:* Data presented are mean ± SD, median (Q1–Q3), median (IQR) or *N* (%).

Abbreviations: BMI, body mass index; CCI score, Charlson comorbidity index; CKD, chronic kidney disease; COPD, chronic obstructive pulmonary disease; GNRI, geriatric nutrition risk index; MI, myocardial infarction; NYHA, New York Heart Association; NT-proBNP, N-terminal pro-B-type natriuretic peptide; SBP, systolic blood pressure.

**Table 2:** Multivariate analysis of readmission of HF patients with different GNRI levels within 6 months.

Variable	HR (95% CI)							
	Crude mode	*p* value	Model 1	*p* value	Model 2	*p* value	Model 3	*p* value
GNRI	0.99 (0.98, 1)	0.007	0.99 (0.98, 1)	0.016	0.99 (0.98, 1)	0.022	0.99 (0.97, 1)	0.038
Tertiles								
GNRI > 98	1 (Ref)		1 (Ref)		1 (Ref)		1 (Ref)	
GNRI ≤ 98	1.19 (1.01, 1.41)	0.038	1.17 (0.99, 1.38)	0.072	1.18 (1, 1.4)	0.056	1.44 (1.08∼1.93)	0.013

*Note:* Model 1: adjusted for gender and age. Model 2: adjusted Model 1 + diabetes, CKD, MI, COPD, NYHA classification, and CCI score. Model 3: adjusted Model 2 + creatinine, uric acid, potassium, sodium, hemoglobin, lactate, brain natriuretic peptide, and diuretics.

## Data Availability

The dataset used in this retrospective cohort study is derived from the publicly accessible Chinese population database PhysioNet, which is available at the following link: https://physionet.org/content/heart-failure-zigong/1.3/. Author Guoxia Dong has successfully completed the educational course provided by the National Institutes of Health and was granted access to the database with certification number 58380637.

## Supporting Information

The supporting information includes the curve fitting results, which are used to further describe the relationship between GNRI and HF 6-month readmission rate.
