# Supplementary figures and images for: The Association Between Geriatric Nutritional Risk Index and Readmission Within Six Months in Elderly Heart Failure Patients: A Retrospective Cohort Study: Geriatric Nutritional Risk Index for Heart Failure Readmission Within 6 Months
Source: Cardiol Res Pract. 2024 Oct 24;2024:5692215. doi: 10.1155/2024/5692215 (PMC11527537; doi:10.1155/2024/5692215)

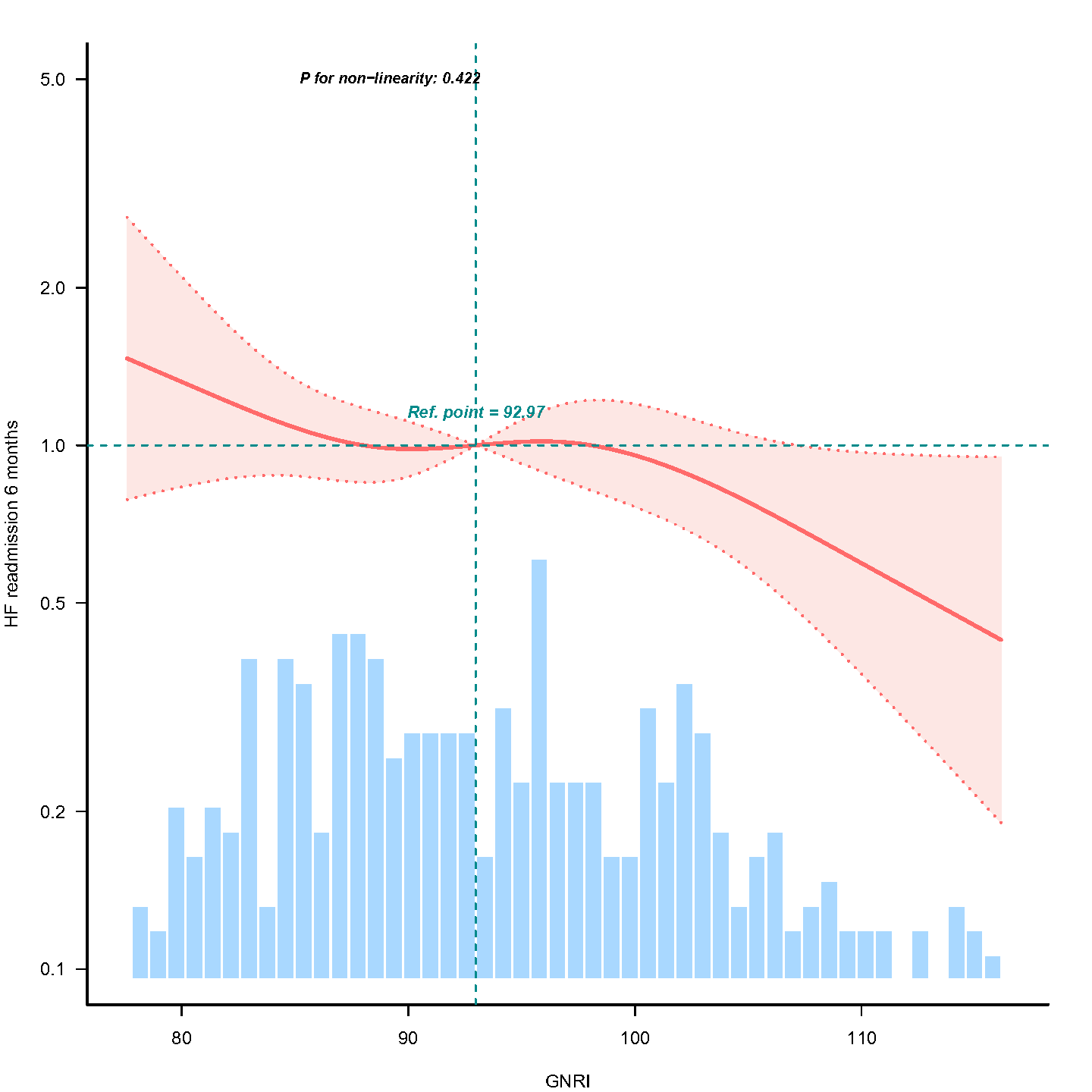


GNRI and HF 6-month readmission rate showed a linear association.

Supplement: Supporting Information — Additional supporting information can be found online in the Supporting Information section. [file 5692215.f1.docx]
